# Predictive analytics characterize cardiovascular function and outcomes following cardiac surgery

**DOI:** 10.1038/s41390-025-04586-w

**Published:** 2025-11-23

**Authors:** Chelsea E. Miller, Sherry L. Kausch, Michael C. Spaeder

**Affiliations:** 1 Department of Pediatrics, University of Virginia School of Medicine, Charlottesville, VA USA; 2 Center for Advanced Medical Analytics, University of Virginia School of Medicine, Charlottesville, VA USA

## Abstract

**Background:**

Using supervised machine learning, predictive models of cardiovascular and respiratory instability were previously created and display risk scores (CoMET score), characterizing the risk of clinical deterioration in the ensuing 12 h. We assessed the relationship of CoMET scores with vasoactive-inotropic scores (VIS) and short-term clinical outcomes in pediatric patients following cardiac surgery.

**Methods:**

Retrospective cohort study of all patients admitted to the pediatric cardiac intensive care unit following cardiac surgery at a single center over an 18-month period. We performed comparative analyses to assess the relationships between CoMET scores and both VIS and postoperative clinical outcomes.

**Results:**

There were 296 postoperative courses in 262 patients during the period of study. Both cardiovascular and respiratory CoMET scores during the first 48 postoperative hours were correlated with VIS (*p* < 0.001). Increased cardiorespiratory CoMET scores during the first 48 postoperative hours were associated with longer postoperative cardiac intensive care unit length of stay (*p* = 0.004), decreased ventilator-free days (*p* < 0.001), and increased incidence of necrotizing enterocolitis (*p* = 0.039), cardiac arrest (*p* = 0.19), and in-hospital mortality (*p* = 0.003).

**Conclusions:**

Display of risk scores of cardiovascular and respiratory instabilities following cardiac surgery may help identify patients at risk for worse clinical outcomes, allowing for earlier intervention, potentially improving outcomes.

**Impact:**

- Using previously established predictive models of risk of cardiovascular and respiratory instability, risk scores were correlated with vasoactive-inotropic scores in pediatric patients immediately following cardiac surgery.
- Increased risk scores following cardiac surgery were associated with increased intensive care unit length of stay, decreased ventilator-free days, and increased incidence of necrotizing enterocolitis, cardiac arrest, and in-hospital mortality.
- Display of risk scores of cardiovascular and respiratory instability following cardiac surgery may help identify patients at risk for worse clinical outcomes, allowing for earlier intervention, potentially improving outcomes.

## Introduction

The past several years have witnessed a rapid rise in the development and adoption of clinical decision support tools, such as predictive analytics, to aid in the identification of patients at risk for clinical deterioration.^1–7^ However, none of these tools have been specifically developed for, or studied in, pediatric patients following cardiac surgery. In the pediatric cardiac intensive care unit (CICU), during the initial period following cardiac surgery, patients are at risk for the development of low cardiac output syndrome (LCOS), which is characterized by inadequate oxygen delivery and a cardiac index less 2.0 L/min/m^2^ that typically occurs 6–18 h post-cardiac surgery in approximately 25% of patients.^8^ This occurs in part due to exposure of the patient’s blood to the cardiopulmonary bypass circuit, which induces a systemic inflammatory response including capillary leak, edema, and myocardial dysfunction.^8^ Close post-operative monitoring and early recognition of LCOS can allow for interventions such as fluid resuscitation and use of vasoactive medications to prevent acute decompensation. Additionally, some patients may be at an increased risk for undesired clinical outcomes such as prolonged postoperative CICU length of stay, increased duration of invasive mechanical ventilation, cardiac arrest, and necrotizing enterocolitis in infants.^9–11^

Using supervised machine learning, we previously developed and implemented predictive models of cardiovascular and cardiorespiratory instability, incorporating real-time telemetric physiologic and laboratory data to display risk estimates for the development of potentially catastrophic clinical events in the subsequent 12 h.^6,7^ Prior work has demonstrated that the vasoactive inotropic score (VIS) can be employed as a surrogate measure of cardiac function in pediatric patients following cardiac surgery.^11^ Our primary aim was to assess for associations between model risk estimates obtained in the first 48 h following cardiac surgery and VIS. Our secondary aim was to assess the association between model risk estimates and CICU morbidity and mortality outcomes.

## Methods

We performed a retrospective study of all patients admitted to the pediatric CICU following cardiac surgery at the UVA Health Children’s Hospital from July 2022 to December 2023. We included patients less than 18 years of age who underwent cardiac surgery with cardiopulmonary bypass as well as infants who underwent pulmonary artery banding, systemic to pulmonary shunting, and coarctation of the aorta repair without bypass. We excluded patients who were admitted to the CICU for less than 48 h or who were admitted to the CICU on mechanical circulatory support. The Institutional Review Board at the University of Virginia School of Medicine approved this study (IRB-HSR #231514) with waiver of consent to analyze routinely collected data. We adhered to the Strengthening the Reporting of Observational Studies in Epidemiology guidelines.^12^

Predictive random forest models of cardiovascular and respiratory instability were previously developed in our CICU.^6,7^ Model-based risk estimates of cardiovascular and respiratory instability are calculated every 15 min incorporating more than three dozen patient-specific variables including: (1) continuous cardiorespiratory waveforms; 2.) vital signs sampled at 0.5 Hz; (3) laboratory values; (4) patient age; and (5) vital sign calculations (e.g., heart rate variability). Full model details are available in the source literature.

**Fig. 1:**
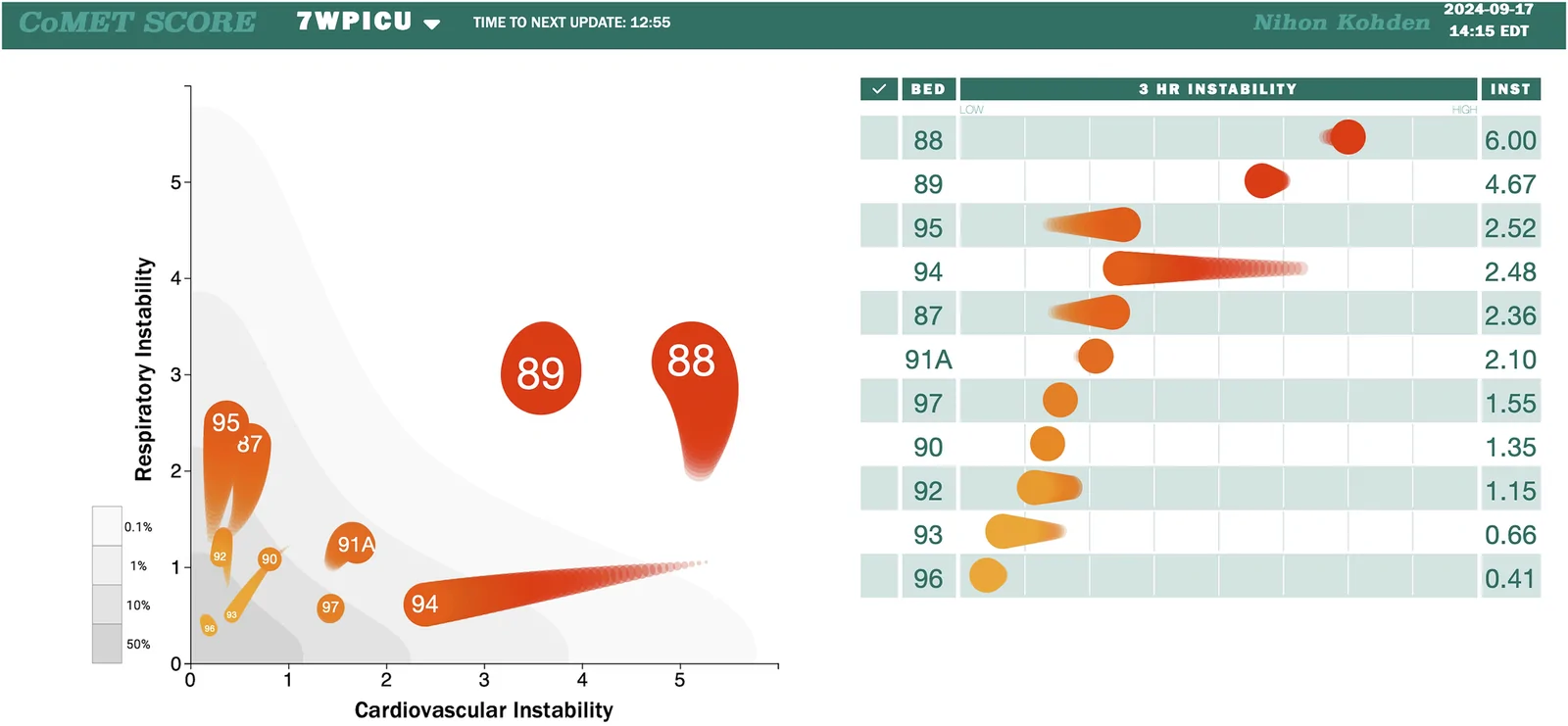
Continuous Monitoring of Event Trajectories (CoMET) Display. Left: Risk estimates, or CoMET scores, are displayed for both the cardiovascular (*x*-axis) and respiratory (*y*-axis) instability domains and represent the fold increase in risk of an event in the next 12 h. A score of 1 means that the risk of an event is at the average risk for the pediatric intensive care unit, with higher numbers meaning higher risk. The bed number is displayed in the head of the CoMET, and the tail represents the score trajectory over the previous 3 h. Right: A composite score of joint risk equal to the square root of the sum of squared cardiovascular and respiratory instability scores is displayed, ordering patients in the cardiac intensive care unit from highest to lowest risk score by room number. The 3-hour tail of the CoMET is also shown to demonstrate the degree of recent stability or instability.

The Continuous Monitoring of Event Trajectories (CoMET^®^) (Nihon Kohden Digital Health Solutions, Irvine, CA) predictive analytics tool displays model risk estimates, or CoMET scores (Fig. 1). These CoMET scores indicate the fold increase in risk as compared to average risk for a patient to develop cardiovascular or respiratory instability in the ensuing 12 h. Additionally, a composite score of joint risk of cardiovascular and respiratory instability scores is displayed, ordering patients in the CICU from highest to lowest CoMET score.

Within the CICU, CoMET scores are displayed on large screen monitors, positioned at the primary work stations. A web-based version of the display is accessible on all computer workstations in the CICU, including bedside computers, and can be accessed on personal digital devices. After validation by the bedside nurse, the average hourly CoMET scores populate within the clinical overview flowsheet of the electronic health record. The clinical response protocol prescribes that an advanced practice provider or physician be informed and assess patients with CoMET scores that have increased by 2 over the previous three hours (e.g., 0.7–2.8) or for a CoMET score greater than 4. We collected baseline demographic and clinical data including age at the time of surgery, birth weight, weight at the time of surgery, sex, type of congenital heart defect including whether the patient had a complete mixing lesion or arch obstruction prior to surgery, type of surgical repair, Society of Thoracic Surgeons-European Association for Cardiothoracic Surgery (STAT) score of surgery, cardiopulmonary bypass time, aortic cross-clamp time, delayed sternal closure, other genetic diagnoses, and presence of heterotaxy. We calculated hourly VIS for the first 48 h following surgery.^11^

We collected data on postoperative clinical outcomes during the index admission, including postoperative ventilator-free days (VFDs) and CICU length of stay, development of necrotizing enterocolitis (NEC), cardiac arrest, and in-hospital mortality. We defined postoperative VFDs as the number of days of invasive mechanical ventilation following surgery to postoperative Day 28; patients who died before Day 28 assigned zero. We employed the modified Bell’s criteria (stage Ib and higher) for the diagnosis of NEC, and the time of the NEC diagnosis was established as the order time of the initiation or broadening of parenteral antibiotics.^13^ We defined a cardiac arrest as any occurrence of cardiopulmonary resuscitation with chest compressions lasting a minimum of 60 seconds.^14^

Our primary aim was to assess the relationship between VIS and average hourly CoMET scores during the first 48 h following surgery by evaluating the relationship between hourly VIS and CoMET cardiovascular and respiratory scores by Spearman cross-correlation at a variety of time lags (zero, one, and two hours). To assess for nonlinear relationships between VIS and hourly CoMET scores, we performed restricted cubic splines transformations using 3 knots of nonlinearity.

We performed linear and logistic regression analyses and reported the area under the receiver operating characteristic (AUC) curve to assess the univariate relationships between the hourly CoMET scores in the first 48 h following surgery and postoperative clinical outcomes. We performed additional analyses to assess the performance of CoMET in the 12 h leading up to a NEC diagnosis or cardiac arrest event. For both NEC diagnosis and cardiac arrest, we identified the CoMET scores in the 12-h window before each event as case data. CoMET scores from all other times in patients with or without events were designated as control data. All data in the 14 days following any event was censored to allow for resolution of NEC or recovery from cardiac arrest. Regression models were constructed to assess for a difference in model output (i.e., risk of event) in the 12 h leading up to an event (case data) as compared to all other times (control data).

We performed all analyses using R Statistical Software (v4.4.1).^15^ Modeling was performed using the *rms* package.^16^ To account for repeated measures, we corrected for unequal variance and correlated measures from individual patients using the Huber-White method to modify the variance-covariance matrix in all regression models.^16^

## Results

The final cohort in this analysis included 296 surgeries in 262 patients. Median age at the time of surgery was 148 days (IQR 17–947). 131 (44.3%) patients had single ventricle anatomy with complete mixing lesions prior to surgery. Within this patient cohort, there were 25 occurrences of necrotizing enterocolitis, and 17 of these occurred in the CICU. The median CICU length of stay was 7 days (IQR: 4–14), and the median number of ventilator-free days was 25 (IQR: 22–28). There were 15 in-hospital mortalities (5.7%). Complete demographic and clinical characteristics are displayed in Table 1.

**Table 1:** Demographic and Clinical Characteristics

Characteristic	*N* (%) or Median (Interquartile Range) (*n* = 296)
Age (months)	5 (IQR 0.5–31)
Male sex	170 (57%)
Heterotaxy	13 (4%)
Single ventricle physiology	131 (44%)
Arch obstruction	60 (20%)
STAT mortality category	
STAT 1	73 (25%)
STAT 2	93 (31%)
STAT 3	60 (20%)
STAT 4	47 (16%)
STAT 5	23 (8%)
CPB time (min)	132 (IQR 81–178)
Cross-clamp time (min)	63 (IQR 0–112)
Delayed sternal closure	29 (10%)
Vasoactive inotropic score	5 (IQR 2.5–7)
Necrotizing enterocolitis	25 (8%)
Cardiac arrest	15 (5%)
Ventilator free days	25 (IQR 22–28)
CICU length of stay (days)	7 (IQR 4–14)
Hospital length of stay (days)	12 (IQR 7–32)

*CPB* cardiopulmonary bypass, *CICU* cardiac intensive care unit, *IQR* interquartile range, *STAT* Society of Thoracic Surgeons – European Association for Cardiothoracic Surgery.

**Fig. 2:**
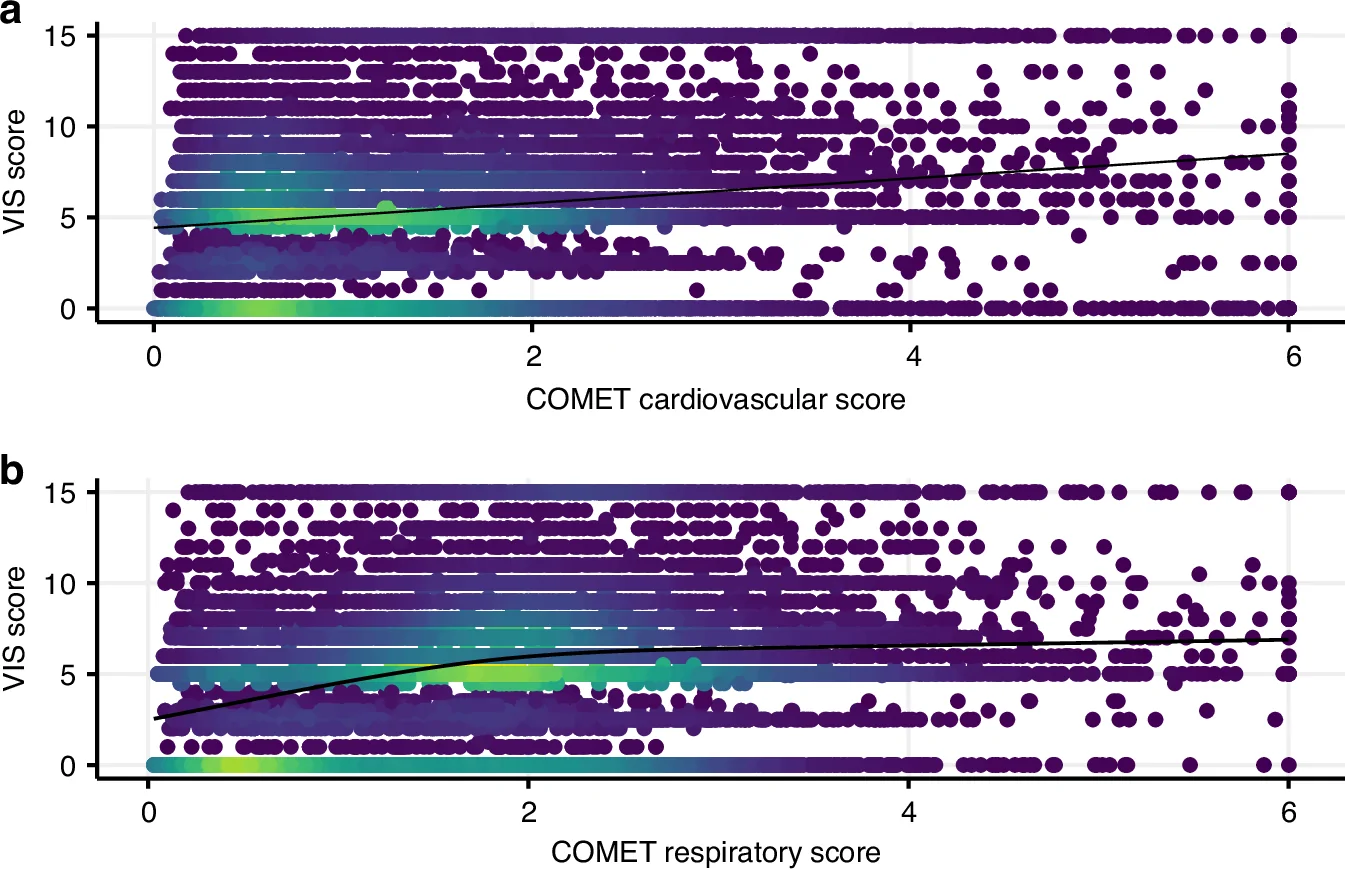
Scatter plot of Continuous Monitoring of Event Trajectories (CoMET) scores and vasoactive-inotrope scores (VIS) in the 48 h following surgery. Plots are displayed for the **a** cardiovascular and **b** respiratory instability components of CoMET. The black line represents the **a** linear and **b** non-linear relationship between the variables. The color saturation represents the density of data points, with lighter colors representing greater density of data.

Hourly cardiovascular and respiratory scores over the first 48 postoperative hours were correlated with VIS with the strongest correlation at time lag zero (both *p* < 0.001). The cross correlation of VIS with cardiovascular scores appeared linear (R = 0.18, R^2^ = 0.032, *p* < 0.001). Respiratory scores were more closely correlated with VIS by restricted cubic splines transformations (R = 0.28, R^2^ = 0.08, *p* < 0.0001) (Fig. 2).

We observed an association between increased CoMET respiratory scores during the first 48 postoperative hours and longer postoperative CICU length of stay (β = 4.08, *p* = 0.004) and decreased VFDs (β=−1.26, *p* < 0.001). Additionally, increased CoMET respiratory scores during the first 48 postoperative hours were associated with an increased incidence of NEC (OR = 1.24 (95% CI 1.01–1.51), *p* = 0.039), cardiac arrest (OR = 1.36 (95% CI 1.05–1.77), *p* = 0.019), and in-hospital mortality (OR = 1.58 (95% CI 1.22–2.05), *p* = 0.003). However, the ability of CoMET respiratory scores during the first 48 postoperative hours to discriminate NEC (AUC = 0.584), cardiac arrest (AUC = 0.612), and in-hospital mortality (AUC = 0.648) was marginal. CoMET cardiovascular scores were not associated with post-operative clinical outcomes.

**Fig. 3:**
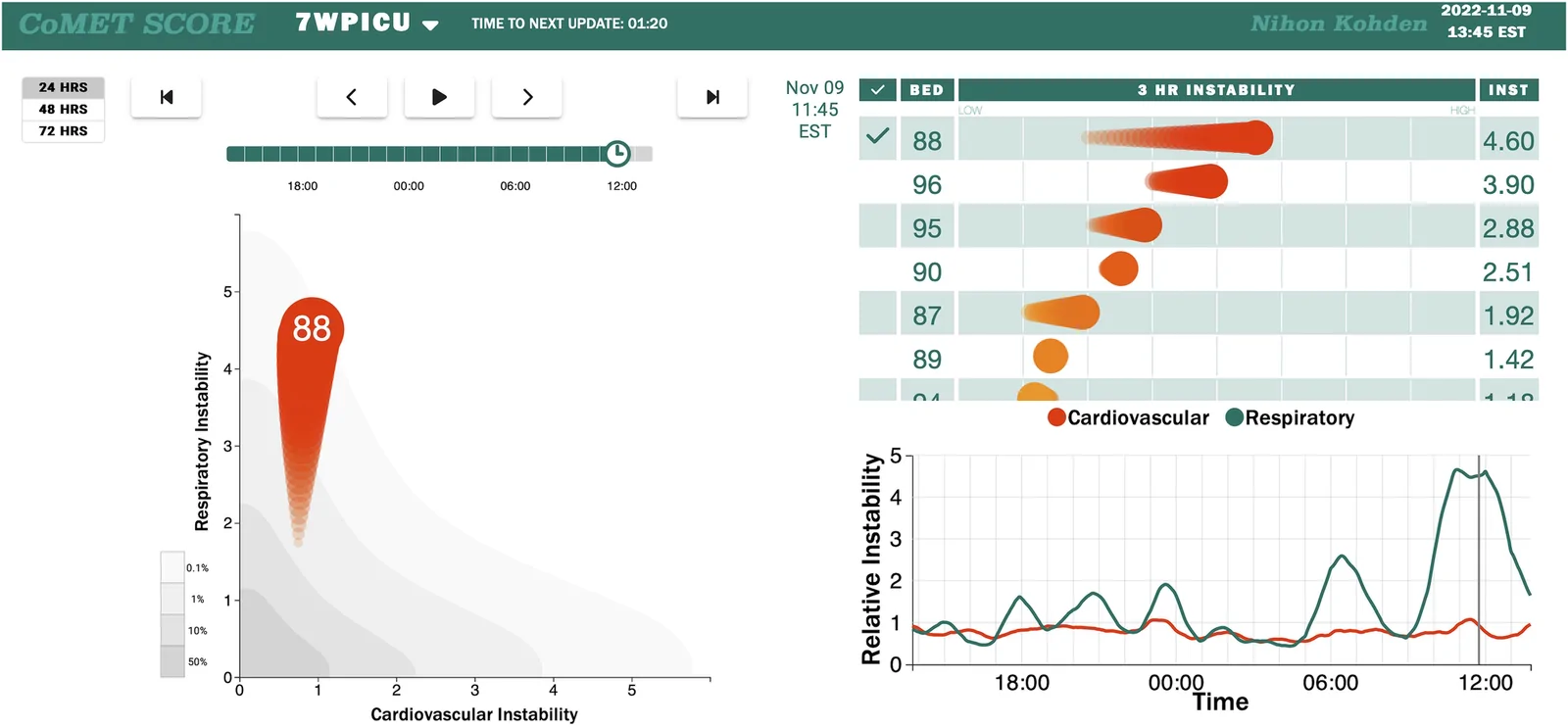
Continuous Monitoring of Event Trajectories (CoMET) case example. Case presentation of a 4-week-old infant with hypoplastic left syndrome following stage 1 palliation and subsequent development of necrotizing enterocolitis (NEC). Right: Specific patients can be chosen for review by selecting the box to the left of their bed number. This action displays the graph shown in the lower right, which is a 24-, 48-, or 72-hour graph showing the progress of the patient’s CoMET scores over a defined period, with cardiovascular instability as the red line and respiratory instability as the green line. Note the escalation in CoMET scores starting around 9 am with the subsequent diagnosis of NEC occurring around 12 pm. Left: The patient’s CoMET is large and bright red at the time of NEC diagnosis, emphasizing the current high level of risk, primarily in respect of their risk of respiratory instability. The tail of the CoMET demonstrates that this instability has markedly increased over the previous 3 h.

There was a significant elevation in CoMET respiratory scores in the 12 h preceding both the diagnosis of NEC (AUC = 0.636, *p* = 0.005) and cardiac arrest (AUC = 0.660, *p* = 0.002). Figure 3 displays the CoMET trajectories of a representative patient in the immediate timeframe leading to the diagnosis of NEC.

## Discussion

We observed that hourly CoMET scores over the first 48 postoperative hours were correlated with VIS, and increased CoMET respiratory scores during this time were associated with worse short-term outcomes. Patients who require higher levels of vasoactive support due to compromised hemodynamics post-operatively are at higher risk for poor outcomes. These patients may require greater fluid resuscitation, which leads to increased edema and, therefore, a longer time to extubation and a need for a longer CICU stay. These patients also experience compromised systemic blood flow immediately post-op, likely from a variety of factors such as an elevated ratio of pulmonary to systemic blood flow, myocardial dysfunction, and obstructive lesions. While this physiology is often improved with significant support in the immediate period, it likely continues to play a role in the development of NEC or cardiac arrest later in the postoperative course. We also observed a significant elevation in CoMET scores in the 12 h prior to diagnosis of NEC and cardiac arrest, which is reflective of this worsening physiology.

Predictive analytics allow for quick acquisition and interpretation of big data that may help detect early signs of clinical deterioration. Specifically, predictive analytics have been used to detect signs of deranged physiology present prior to clinical detection of potentially catastrophic illnesses in the intensive care unit setting.^17^ This technology may help identify patients at highest risk, enabling earlier intervention and better outcomes.

The use of predictive analytics has become more prevalent in pediatric intensive care over the past few years.^2^ However, a small percentage of predictive models have been implemented or undergone external validation.^2^ While finding ways to predict poor outcomes such as cardiac arrest is an important first step, the clinical impact of such models will come from the utilization and prevention of such outcomes. Dewan and colleagues investigated the effectiveness of the Inadequate Oxygen Delivery Index (IDO_2_; Etiometry, Cambridge, MA) in pediatric patients following cardiac surgery to identify patients at high risk for cardiac arrest.^5^ Following implementation of CoMET in our CICU, we observed an improvement in cardiac arrest outcomes, most notably a significant increase in the rate of return of spontaneous circulation as compared to the pre-CoMET implementation time period.^14^ As this study has added to the body of evidence that CoMET may aid in the detection of hemodynamic instability and predict poor outcomes in pediatric patients after cardiac surgery, continued implementation and clinical utilization should be assessed for improving important patient outcomes.

We were initially struck by the observation that the CoMET respiratory score performed better than the cardiovascular score in all the assessed clinical associations. While labeled as the cardiovascular score by the manufacturer, the model was trained and validated on the outcome of sepsis in a cohort of patients in a combined cardiac and medical-surgical pediatric intensive care unit.^6^ While there is obvious physiologic and biochemical overlap between sepsis and LCOS captured by the cardiovascular instability model (e.g., tachycardia, metabolic acidosis), we surmise that there was insufficient variability in other important features of sepsis prediction (e.g., age, white blood cell count, temperature) amongst post-operative cardiac surgical patients to reliably discriminate for cardiac dysfunction. Interestingly, during the construction of the respiratory instability model, we noted very distinct differences in the signatures of illness for respiratory failure (defined as urgent unplanned intubation) between cardiac surgical patients and both medical and non-cardiac surgical patients.^7^ Medical and non-cardiac surgical patients typically experienced a primary respiratory failure (e.g., oxygenation and/or ventilation failure) whereas cardiac surgical patients were more like to exhibit hemodynamic instability necessitating intubation.^7^ We hypothesize that it is this distinction that explains why the CoMET respiratory score performs better amongst cardiac surgical patients in its correlation with VIS and discrimination of clinical outcomes.

An important distinction to highlight is that CoMET was designed and implemented as a trajectory-based tool to provide a visual representation of a patient’s clinical trajectory through the real-time analysis of physiologic and biochemical data. While we employ a clinical response protocol in our CICU, we cannot objectively characterize the process and the balancing metrics regarding protocol adherence or alert burden. Over the period of study, 5.5% of the time one or both risk scores exceeded 4, equating to 11 instances per day.

Additional limitations of this study must be considered, including the magnitude of the correlation between CoMET scores and VIS. This was a single-center study, and the management of pediatric patients after cardiac surgery likely varies significantly between centers, making generalizability unclear. As CoMET scores were already displayed in the CICU at our institution, it is possible that providers intervened in response to these scores during the stud,y which we cannot account for. We could not account for post-operative interventions such as fluid or sedative administration that may impact hemodynamic status, though this is likely worthy of future study. Finally, while calculated every 15 min incorporating the previous 30 min of vital signs, CoMET scores are archived as hourly averages and thus, we may have been unable to detect a more granular time-dependent relationship between VIS and CoMET scores.

## Conclusions

We observed that a predictive analytics model, CoMET, was correlated with VIS after cardiac surgery and predicted poor short-term outcomes. The use of such models may aid in earlier detection of LCOS and possibly prevention of acute decompensation.

## Data Availability

The datasets presented in this article are not readily available because the conditions of institutional approval do not provide for the transmission of datasets outside of the sponsoring institution. Requests to access the datasets should be directed to ms7uw@uvahealth.org.
